# Fisetin-loaded nanoemulsion ameliorates lung cancer pathogenesis via downregulating cathepsin-B, galectin-3 and enolase in an *in vitro* setting

**DOI:** 10.17179/excli2024-7583

**Published:** 2024-10-14

**Authors:** Ayeh Bani Saeid, Keshav Raj Paudel, Gabriele De Rubis, Samir Mehndiratta, Sofia Kokkinis, Sukriti Vishwas, Stewart Yeung, Gaurav Gupta, Sachin Kumar Singh, Kamal Dua

**Affiliations:** 1 Discipline of Pharmacy, Graduate School of Health, University of Technology Sydney, Ultimo, NSW, 2007, Australia; 2 Faculty of Health, Australian Research Center in Complementary and Integrative Medicine, University of Technology Sydney, Ultimo, NSW, 2007, Australia; 3 Center for Inflammation, Faculty of Science, School of Life Sciences, Centenary Institute and University of Technology Sydney, Sydney, NSW, 2007, Australia; 4 School of Pharmaceutical Sciences, Lovely Professional University, Jalandhar-Delhi G.T Road, Phagwara, 144411, India; 5 Center for Research Impact & Outcome, Chitkara College of Pharmacy, Chitkara University, Punjab, India; 6 Center of Medical and Bio-allied Health Sciences Research, Ajman University, Ajman, United Arab Emirates

## ⁯

Lung cancer (LC) remains one of the leading causes of cancer-related deaths worldwide, with a high incidence and mortality rate (Barta et al., 2019[6]). According to the World Health Organization (WHO), in 2020 alone, around 2 million LC diagnoses and 1.8 million deaths worldwide were recorded, showing its great burden on public health (WHO, 2023[53]). LC was also the fifth most frequently diagnosed cancer in Australia in 2019, with 13,140 new cases reported that year (Cancer Australia, 2019[7]). Lung cancer often stems from a complex interplay of genetic predisposition, environmental exposures, and lifestyle choices (Travis, 2020[48]). Statistics reveal that smokers are 15 to 30 times more likely to develop lung cancer than non-smokers, with nearly 90 % of lung cancer deaths in men and 80 % in women linked to smoking (Alharbi et al., 2022[1]; Chang et al., 2021[9]). Moreover, exposure to second-hand smoke increases the risk by 20-30 % (Chang et al., 2021)[9]. Furthermore, genetic predispositions, including inherited mutations, play a role, although they are less common (Travis, 2020[48]). Hence, lung cancer's pathogenesis is multifaceted, driven by genetic mutations, epigenetic alterations, and dysregulated signaling pathways, including but not limited to EGFR, RAS/MAPK, PI3K/AKT/mTOR, JAK/STAT, and Wnt/β-catenin, promotes tumor growth and survival (Alharbi et al., 2022[2]; Koni et al., 2020[21]; Mehta et al., 2020[31]). In addition to these signaling pathways and the proteins involved in them, there are some other proteins whose functions, as indicated by some studies, can indirectly play important roles in the progression of lung cancer, including cathepsin B (CTSB), enolase, and galectin-3 (GAL3) (Gondi and Rao; 2013[11], Huang et al., 2017[18]; Pokhare et al., 2022[35]).

Understanding these molecular mechanisms provides insights into potential therapeutic targets and strategies for personalized medicine in lung cancer treatment (Alnuqaydan et al., 2022[3][4]; Singh et al., 2024[42]). Current lung cancer treatments, such as chemotherapy, radiation therapy, and targeted therapy, often face limitations like resistance to treatment, reducing their long-term effectiveness (Hirsch et al., 2017[16]; Saeid et al., 2023[39]). Additionally, severe side effects can significantly impact patients' quality of life (Hirsch et al., 2017[16]). One of the treatment options that has attracted researchers' attention in recent years is fisetin, a naturally occurring flavonoid found in various fruits and vegetables such as strawberries, apples, and onions (Ling et al., 2022[27]; Paudel et al., 2022[33]; Prasher et al., 2020[36]; Saeid et al., 2023[39]). Fisetin has garnered attention for its wide array of biological activities, including anti-inflammatory, antioxidant, and anticancer properties (Imran et al., 2021[20]; Ling et al., 2022[27]). Its anticancer activity is linked to its regulation of key signaling pathways like PI3K/AKT, mTOR, and Wnt/β-catenin, as well as its capacity to induce apoptosis and inhibit angiogenesis (Imran et al., 2021[20]). Despite its promising therapeutic potential, the clinical application of fisetin is often limited by its poor bioavailability and rapid metabolism due to its poor water solubility, which necessitates strategies to enhance and prolong its circulation time for optimal therapeutic efficacy (Szymczak and Cielecka-Piontek, 2023[45]). 

Given these challenges, nano emulsions, classified as colloidal dispersions consisting of two immiscible liquids, offer a novel solution to enhance the delivery and efficacy of fisetin (Kumar et al., 2023[25]). They form transparent dispersions of oil in water or water in oil with particle sizes typically ranging from 20 to 200 nm, stabilized by surfactants (Ranjbar et al., 2023[38]; Szymczak and Cielecka-Piontek, 2023[45]). More recently, self-nanoemulsifying drug delivery systems (SNEDDS) have emerged as anhydrous nano emulsions composed of oil, a surfactant, a co-surfactant, and a drug molecule, which, upon oral administration, form nano emulsions in the stomach (Liu et al., 2021[28]; Sharma et al., 2019[41]; Vishwas et al., 2022[50]). This smaller particle size in the nano range increases the surface area for absorption, thus enhancing drug bioavailability (Vishwas et al., 2022[50]). These advantages have prompted researchers to explore the use of self-nanoemulsifying drug delivery systems, including fisetin, to improve its solubility, permeability, and bioefficacy (Kumar et al., 2022[24]; Vishwas et al., 2022[50]). Given the intricate role of CTSB, enolase, and GAL3 enzymes in lung cancer progression and the potential benefits of fisetin, this study aims to explore the effectiveness of a fisetin-loaded nano emulsion (FNE) in attenuating these pathological markers, *in vitro*, in lung cancer cells, specifically the A549 human lung adenocarcinoma cells. We show that the FNE significantly downregulates CTSB, enolase, and GAL3 enzymes compared to the control group.

 The SNEDDS formulation for fisetin (FS-SNEDDS) was prepared by combining specific amounts of castor oil (0.1 mL), Lauroglycol FCC (0.1 mL), Tween 80 (0.4 mL), and Transcutol P (0.6 mL) in a clean glass vial. These components were thoroughly mixed before adding fisetin (5 mg). The formulation and evaluation processes were based on the methods previously detailed by Kumar et al (Kumar et al., 2019[23], 2022[24]). A549 lung cancer cells were cultured in a humidified environment with 5 % CO_2_ at 37 °C. The medium was refreshed every 48 hours. Cells were plated at a density of 250,000 cells per well in six-well plates and treated with 10 µg/mL fisetin nano emulsion (FNE) for 24 hours the following day. Notably, the non-cytotoxic concentration of 10 µg/mL was determined through an MTT (3-(4,5-Dimethylthiazol-2-yl)-2,5-diphenyltetrazolium bromide) cell viability assay, which indicated that the minimum cytotoxic concentration of FNE in A549 cells was 20 µg/mL (data not shown). After treatment, the medium was removed, and cells were washed with ice-cold phosphate-buffered saline (PBS). Cells were lysed using a radioimmunoprecipitation assay (RIPA) buffer containing protease inhibitor tablets, and the mixture was incubated on ice for 15 minutes. The lysate was then centrifuged at 4 °C for 15 minutes at 15,000 g to remove debris. The supernatant was collected for protein concentration determination using the Pierce™ BCA Assay Kit. The influence of FNE on cancer-related protein expression was analyzed using the Proteome Profiler Human XL Oncology Array Kit. Each array membrane was loaded with 300 µg of protein, hybridized, processed, and imaged using a ChemiDoc™ MP imaging system. Pixel intensity for each protein was measured using ImageJ software, and statistical analysis was performed using PRISM software (Paudel et al., 2024[34]).

The results of this study demonstrate that treating A549 cells with 10 µg/mL FNE can significantly reduce the expression levels of three key proteins involved in the pathogenesis of lung cancer, specifically in cancer invasion and metastasis: CTSB, enolase, and GAL3. While some studies have already explored the link between fisetin and its effect on these markers (Hsieh et al., 2019[17]; Kumar et al., 2022[24]; Malathi et al., 2022[29]; Singh et al., 2018[43]; Zhang and Cui, 2021[56]), only a limited number of *in vitro* studies have been conducted on lung cancer in this context, making the present study unique. For instance, GAL3, a protein encoded by the *LGALS3* gene, is known to play diverse roles in cancer initiation, progression, and drug resistance, as discussed in an earlier section (Capone et al., 2021[8]; Zhang et al., 2021[55]). Kusuhara et al. (2021[26]) demonstrated that the high GAL3 protein expression in tumor cells is associated with poor prognosis in non-small cell lung cancer (NSCLC) (Kusuhara et al., 2021[26]). Moreover, GAL3 has been identified as a predictive factor for recurrence-free survival (RFS) in NSCLC patients receiving platinum-based adjuvant chemotherapy (AC) (Kusuhara et al., 2021[26]). Our observation of decreased GAL3 expression following FNE treatment (Supplementary information, Figure 1c) aligns with the notion that targeting GAL3 may hold therapeutic promise in lung cancer management (Kusuhara et al., 2021[26]; Torres‐Martínez et al., 2024[47]). In addition, squamous cell carcinoma of the lung is recognized as an aggressive disease with a poor prognosis. While the majority of patients do not survive longer than five years, a minority show prolonged survival without disease progression (Pokhare et al., 2022[35]). According to Pokhare and colleagues (2022[35]), the identification of biomarkers, such as GAL3, using easily available immunohistochemical assays could improve risk stratification in lung cancer patients (Pokhare et al., 2022[35]), highlighting the importance of considering this biomarker even more in future studies.

Research has also shown that most malignant cells can increase the transcriptional activation of CTSB, an intracellular catabolic protein involved in the processing of antigens in the immune response. For instance, Sloane et al. (1981[44]) have indicated a strong connection between the activity of CTSB and the aggressive metastatic nature of pancreatic cancer and melanoma (Sloane et al., 1981[44]). In pancreatic cancer, CTSB is crucial in fostering tumor growth, angiogenesis, and invasion (Tzanakakis et al., 2003[49]). It is speculated that Matrix Metalloproteinases (MMPs), enzymes that break down proteins in the extracellular matrix (ECM), are activated or shed into the ECM via the action of CTSB and urokinase-type plasminogen activator (uPA). Essentially, CTSB is crucial in managing the protease cascade and guiding it to the invasive edges of metastatic cells (Gondi and Rao, 2013[11]). This pattern of elevated CTSB expression has also been reported for lung cancer, where it is associated with increased tumor aggressiveness and poorer patient prognosis, potentially hindering the prediction of patients' response to chemotherapy (Gormley et al., 2011[12]; Harbeck et al., 2001[14]; Werle et al., 1999[52]). Moreover, a molecular docking study analyzed the interaction of fisetin with different immune-related proteins and biomarkers, concluding that CTSB had one of the strongest predicted interactions with fisetin, exhibiting the lowest binding energy (Malathi et al., 2022[29]). The latter two points, (i) the relevance of CTSB in lung cancer, and (ii) the potential of fisetin in interacting with CTSB, highlight the significance of the results of the present study (Supplementary information, Figure 1a), suggesting the potential of fisetin as suitable treatment for lung cancer through targeting the CTSB expression level.

Furthermore, the role of enolase, an enzyme catalyzing an important glycolytic pathway reaction, has been extensively linked to lung cancer, as discussed in an earlier section (Schofield et al., 2020[40]; Tian et al., 2020[46]). However, this role is primarily in LC diagnosis as a preventive value (Xu et al., 2019[54]). According to many studies, evaluating the expression of enolase aids in lung cancer diagnosis, distinguishing between different subtypes, and assessing tumor characteristics (Fujita et al., 1987[10]; Huang et al., 2017[18]; Kostovski and Petrushevska, 2014[22]; Wang et al., 2016[51]). Serum enolase levels correlate with tumor size, stage, and metastasis in small-cell lung cancer (SCLC) (Huang et al., 2016[19]). Furthermore, enolase levels serve as a sensitive marker for monitoring therapy and predicting relapse, guiding treatment decisions (Hirose et al., 2011[15]; Nitta et al., 1995[32]). With substantial evidence highlighting the significance of enolase in lung cancer, therapy strategies are now considering targeting this biomarker to reduce its levels, especially in SCLC (Tian et al., 2020[46]). A recent study found that fisetin can trigger apoptosis in cancer cells through multiple pathways, many of which are supported by enolase activity. These pathways include promoting proteasomal degradation and reducing the half-life of specific proteins, deactivating receptors, decreasing enolase phosphorylation, and modifying PI3K/AKT signaling (Guo et al., 2019[13]). Inhibiting enolase activity could disrupt these metabolic pathways that support tumor growth and metastasis (Rahmani et al., 2022[37]), offering a potential strategy for lung cancer treatment. This comprehensive logic behind the role of enolase in lung cancer and the possible effect of fisetin in targeting it aligns with the results (shown in Supplementary information, Figure 1b), where the expression level of enolase in A549 cells significantly decreased after treatment with FNE.

Although the result of the present study shows that 10 µg/mL of fisetin nano emulsion can significantly reduce the expression levels of relevant markers such as CTSB, enolase, and GAL3 in lung cancer cells, we also have several limitations to consider. The study's reliance on an *in vitro* model restricts the extrapolation of these findings to *in vivo* conditions, where factors such as metabolism, immune response, and tissue-specific interactions may influence treatment outcomes differently (Antunes et al., 2022[5]; Malyla et al., 2020[30]). Furthermore, while the investigation targeted key markers associated with lung cancer progression, the complexity of lung cancer's molecular pathways suggests that other important biomarkers and pathways may not have been comprehensively examined. Therefore, further research employing diverse experimental models and clinical trials is essential to validate these results and ascertain fisetin nano emulsions broader therapeutic potential and safety profile in lung cancer treatment.

## Notes

Ayeh Bani Saeid and Keshav Raj Paudel contributed equally as first author.

Sachin Kumar Singh and Kamal Dua (Discipline of Pharmacy, Graduate School of Health, University of Technology Sydney, Ultimo, NSW, 2007, Australia; E-mail: kamal.dua@uts.edu.au) contributed equally as corresponding author.

## Declaration

### Acknowledgments

The authors are thankful to the Graduate School of Health, University of Technology Sydney, Australia. KD, GDR, and KRP are supported by the Maridulu Budyari Gumal Sydney Partnership for Health, Education, Research and Enterprise (SPHERE) RSEOH CAG and Triple I Seed Grant 2023 and Extension Grant, Triple I Skills Advancement Grants 2023 and 2024, and the UTS Faculty of Health Industry Partnership Grant. GDR is also supported by the UTS Faculty of Health Category 1 Seeding grants for Early Career Researchers.

### Declaration of competing interest 

The authors declare that there is no conflict of interest regarding the publication of this letter.

## Supplementary Material

Supplementary information
