# Supplementary material for: Fisetin-loaded nanoemulsion ameliorates lung cancer pathogenesis via downregulating cathepsin-B, galectin-3 and enolase in an in vitro setting
Source: EXCLI J. 2024 Oct 14;23:1238–44. doi: 10.17179/excli2024-7583 (PMC11579513; doi:10.17179/excli2024-7583)
Supplement: Supplementary information [file EXCLI-23-1238-s-001.pdf]

EXCLI Journal 2024;23:1238-1244 – ISSN 1611-2156
Received: July 13, 2024, accepted: October 09, 2024, published: October 14, 2024

## Supplementary information to:

## Letter to the editor:

# FISETIN-LOADED NANOEMULSION AMELIORATES LUNG CANCER PATHOGENESIS *VIA* DOWNREGULATING CATHEPSIN-B, GALECTIN-3 AND ENOLASE IN AN *IN VITRO* SETTING

Ayeh Bani Saeid[a,b,#], Keshav Raj Paudel[c#], Gabriele De Rubis[a,b], Samir Mehndiratta[a], Sofia Kokkinis[a,b], Sukriti Vishwas[d], Stewart Yeung[a,b], Gaurav Gupta[e,f], Sachin Kumar Singh[b,d,*], Kamal Dua[a,b,*]

[a] Discipline of Pharmacy, Graduate School of Health, University of Technology Sydney, Ultimo, NSW, 2007, Australia
[b] Faculty of Health, Australian Research Center in Complementary and Integrative Medicine, University of Technology Sydney, Ultimo, NSW, 2007, Australia
[c] Center for Inflammation, Faculty of Science, School of Life Sciences, Centenary Institute and University of Technology Sydney, Sydney, NSW, 2007, Australia
[d] School of Pharmaceutical Sciences, Lovely Professional University, Jalandhar-Delhi G.T Road, Phagwara, 144411, India
[e] School of Pharmacy, Graphic Era Hill University, Dehradun, 248007, India
[f] Department of Clinical Sciences, College of Pharmacy and Health Sciences, Ajman University, Ajman, United Arab Emirates

# These authors contributed equally as first authors.

* **Corresponding authors**: Kamal Dua, Discipline of Pharmacy, Graduate School of Health, University of Technology Sydney, Ultimo, NSW, 2007, Australia.
E-mail: kamal.dua@uts.edu.au
Sachin Kumar Singh, School of Pharmaceutical Sciences, Lovely Professional University, Jalandhar-Delhi G.T Road, Phagwara, 144411, India. E-mail: sachin.16030@lpu.co.in; singhsachin23@gmail.com

https://dx.doi.org/10.17179/excli2024-7583

This is an Open Access article distributed under the terms of the Creative Commons Attribution License (http://creativecommons.org/licenses/by/4.0/).

## METHODS

The liquid SNEDDS formulation comprised castor oil (0.1 mL), Lauroglycol FCC (0.1 mL), tween 80 (0.4 mL), and Transcutol P (0.6 mL). These components were combined in the specified amounts in a clean glass vial and thoroughly mixed, after which fisetin (5 mg) was added to create the FS-SNEDDS. The entire process of formulating and evaluating FS-SNEDDS was detailed in previous studies conducted by Kumar et al. (Kumar et al., 2019, Kumar et al., 2022).

A549 cells were cultured in a humidified environment with 5 % $CO_2$ at 37 °C, and the culture medium was regularly refreshed every 48 hours. The cells were initially plated at a density of 250,000 cells per well in six-well plates and, on the next day, were treated with a specified

EXCLI Journal 2024;23:1238-1244 – ISSN 1611-2156
Received: July 13, 2024, accepted: October 09, 2024, published: October 14, 2024

concentration (10 µg/mL) of FNE for a duration of 24 hours. The medium was removed after the treatment, and the cells were washed with ice-cold phosphate-buffered saline (PBS, Merck, Australia). Subsequently, the cells were lysed by adding a solution consisting of radioimmuno-precipitation assay (RIPA) buffer (ThermoFisher, Australia) supplemented with protease inhibitor tablets (Merck, Australia), and the mixture was incubated on ice for 15 minutes to ensure complete cell lysis. To remove any solid residues and cellular debris, the lysate was centrifuged at 4 °C for 15 minutes at 15,000 g. The resulting supernatant, devoid of debris, was collected, and its protein concentration was determined using a Pierce™ BCA Assay Kit (ThermoFisher Scientific, Australia). To analyze how FNE influences cancer-related protein expression, the Proteome Profiler Human XL Oncology Array Kit was employed. Each membrane was loaded with 300 µg of proteins. Subsequently, the array membranes were hybridized and processed as specified by the product manufacturer, then imaged using a ChemiDoc™ MP imaging system from BioRad (Australia). Pixel intensity for each protein was measured using ImageJ version 1.53c (Bethesda, USA), and statistical analysis was carried out using PRISM version 9.3 (GraphPad, USA) (Paudel et al., 2024).

## RESULTS

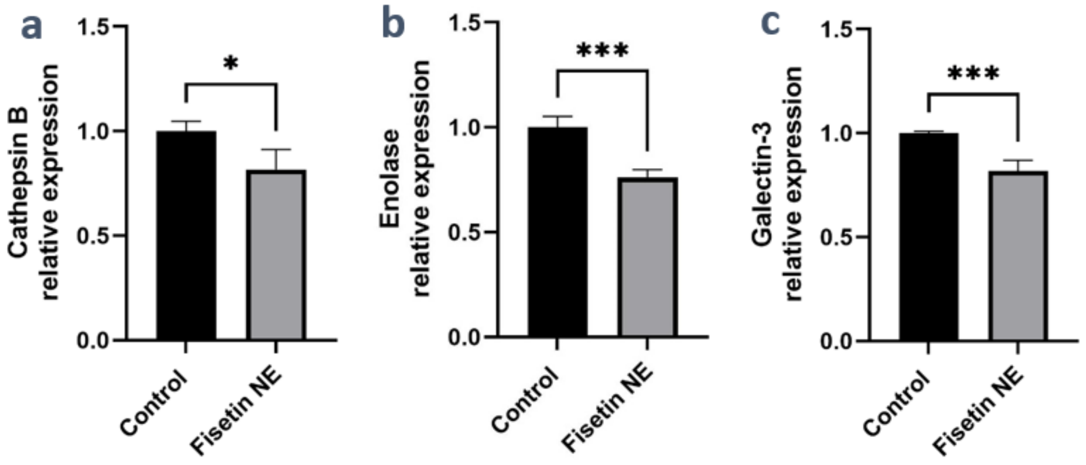


**Supplementary Figure 1:** FNE downregulates the expression of lung-cancer-related markers on A549 human lung adenocarcinoma cells *in vitro*. A549 human lung adenocarcinoma cells were subjected to either treatment with 10 µg/mL FNE for 24 hours or left untreated. Cell lysates were prepared using RIPA buffer, and 300 µg of extracted proteins from each group were analyzed using the Proteome Profiler Human XL Oncology Array Kit. The chemiluminescence, expressed as pixel density, was quantified with ImageJ software. Data are presented as mean ± SEM (n = 4). Statistical analysis was performed using Unpaired Student's t-test, where significance levels are denoted as * for P<0.5 and *** for P<0.001. The expression level of CTSB was downregulated by 20 % **(Figure 1a)**, while this percentage was higher for enolase **(Figure 1b)** and GAL3 **(Figure 1c)**, with reductions of 25 % and 22 %, respectively.

## REFERENCES

Kumar R, Khursheed R, Kumar R, Awasthi A, Sharma N, Khurana S, et al. Self-nanoemulsifying drug delivery system of fisetin: Formulation, optimization, characterization and cytotoxicity assessment. J Drug Deliv Sci Technol. 2019;54:101252. doi: 10.1016/j.jddst.2019.101252.

Kumar R, Kumar R, Khurana N, Singh SK, Khurana S, Verma S, et al. Improved neuroprotective activity of Fisetin through SNEDDS in ameliorating the behavioral alterations produced in rotenone-induced Parkinson's model. Environ Sci Pollut Res. 2022; 29:50488-99. doi: 10.1016/j.jddst.2019.101252.

*EXCLI Journal 2024;23:1238-1244 – ISSN 1611-2156*
*Received: July 13, 2024, accepted: October 09, 2024, published: October 14, 2024*

Paudel KR, Mohamad MSB, De Rubis G, Reyes R-J, Panth N, Dureja H, et al. 18-β-Glycyrrhetinic acid encapsulated PLGA nanoparticles attenuate lung cancer proliferation and migration. J Drug Deliv Sci Technol. 2024;95:105523. doi: 10.1016/j.prp.2024.155295.
